# Editorial: Hybrid effectiveness-implementation trial designs—critical assessments, innovative applications, and proposed advancements

**DOI:** 10.3389/frhs.2024.1414969

**Published:** 2024-05-17

**Authors:** Bo Kim, Marcel Yotebieng, Ann Catrine Eldh

**Affiliations:** ^1^ Center for Healthcare Organization and Implementation Research, VA Boston Healthcare System, Boston, MA, United States; ^2^ Department of Psychiatry, Harvard Medical School, Boston, MA, United States; ^3^ Division of General Internal Medicine, Department of Medicine, Albert Einstein College of Medicine, Bronx, NY, United States; ^4^ Department of Health, Medicine, and Caring Sciences, Linköping University, Linköping, Sweden; ^5^ Department of Public Health and Caring Sciences, Uppsala University, Uppsala, Sweden

**Keywords:** hybrid effectiveness-implementation studies, knowledge translation, implementation science, study designs, mixed-methods research

**Editorial on the Research Topic** Hybrid effectiveness-implementation trial designs—critical assessments, innovative applications, and proposed advancements

## 1 Introduction to hybrid studies and this Research Topic

Hybrid effectiveness-implementation studies contribute to accelerating the translation of evidence into practice, for the benefit of safer and better healthcare services. Such studies examine the effectiveness of an innovation alongside its implementation. Since their codification in 2012 by Curran and colleagues, hybrid studies have become increasingly prevalent in implementation research.

The growing collection of hybrid studies is bringing to light various combinations of data sources and research methods that the studies comprise. Reports of these studies demonstrate both the flexibility with which study teams have approached co-examining effectiveness and implementation, as well as the need for further guidance on making design decisions for hybrid studies.

This article provides an overview of hybrid effectiveness-implementation studies, their importance for implementation science across various healthcare contexts, and their applications to date. It then points the reader to key considerations for furthering the impact of hybrid studies, which are emphasized by the articles included in this Research Topic of Frontiers in Health Services.

## 2 Hybrid studies’ role in implementation science and future directions

Implementation science focuses on growing the knowledge base of how to more rapidly, efficiently, and sustainably translate evidence into practice. Grounded in both theories of change and historical examples of there being a lag between an innovation being shown to be effective and its uptake into routine practice, it has been adopted by healthcare researchers recognizing its potential to address the know-do gaps in their services. Originally, implementation research was conceptualized as taking place once prior research has established an innovation's effectiveness. Under this conceptualization, implementation research (i) identifies barriers and enablers of implementing innovations and (ii) develops, tests, and refines strategies to address the barriers and leverage the enablers in successfully implementing the innovations.

Hybrid effectiveness-implementation studies modify such original conceptualization to decrease the evidence-to-practice lag even more. Namely, they prepare for an innovation's implementation sooner by studying implementation barriers, enablers, and strategies while the innovation's effectiveness is still being examined. The goal of effectiveness studies has always been to investigate whether an innovation works in a real-world setting, requiring the innovation to be implemented for its effectiveness to be studied. Integrating the implementation assessment component takes advantage of this requirement, expanding the investigation to include the contexts and actions under which the innovation works (or does not work), for whom, and why (or why not).

Expanding the scope of investigation to include both effectiveness and implementation is not a simple task. There are ethical considerations for expending resources on an innovation that is yet to be shown to be effective. And as in the case of any study design, hybrid studies require well-justified trade-offs between expanding and limiting the scales of its effectiveness and implementation investigations that are pursued with limited resources (e.g., funding, time, personnel). Importantly, these considerations are affected by the complexity of real-world settings in which the studies are conducted, which host myriad factors at individual, organizational, and societal levels that could influence processes and thus affect study outcomes. This calls for further means to comprehend and illuminate study findings. While many studies are embracing mixed methods to better understand these factors, there is a need for additional thoroughness to validly plan and execute hybrid inquiries.

As hybrid studies enter their second decade of widespread use, needed is a research agenda to meaningfully curate improved operationalizations and continued relevance of hybrid studies in catalyzing timely implementation of effective innovations. First, critical assessments of past hybrid studies would elucidate their heterogeneous characterizations and extents of success. Second, innovative applications of research methods in current hybrid studies would point to more appropriate and, potentially, more efficient concurrent investigation of effectiveness and implementation. Third, proposed advancements for use in future hybrid studies would establish the tasks needed to enhance the field's knowledge of hybrid studies, especially regarding whether the studies actually speed the translation of research evidence into routine healthcare practice and how. Crucially, each of these items should take into account contemporaneous and dynamically progressing foci of implementation research—e.g., decrease in health disparities through equitable implementation, modifications to innovations and implementation strategies to meet contextual needs, and economic evaluations that inform implementation efforts.

## 3 Overview of this Research Topic

As a first step towards collaboratively and inclusively developing a research agenda for hybrid studies, this Research Topic is meant to fuel the field's discourse on the past, present, and future of hybrid effectiveness-implementation studies. To enable a discourse that is both rooted in expertise while inviting of new perspectives, the included articles address a broad audience of healthcare researchers, practitioners, educators, clients, and policymakers that shape implementation science.

The articles cover both specific examples of hybrid studies and syntheses of trends across studies, putting forward novel considerations to help better assess, apply, and advance hybrid study designs for concurrently examining innovations’ effectiveness and implementation. Specifically, the articles are:

• Garner’s article discusses examples of hybrid studies that lie across the effectiveness-to-implementation continuum, and proposes an approach to explicitly incorporate dissemination-, sustainment-, economics-, and scaling-related considerations into designing hybrid studies.
• Jurczuk and colleagues’ article challenges the field to improve the thought and rigor applied to selecting comparison groups for hybrid studies testing an innovation’s effectiveness and implementation, through narratively reviewing how trial-based studies have designed and managed their control arm.
• Grice-Jackson and colleagues’ article shares process evaluation findings from a hybrid mixed-methods study aimed at better assessing and reducing cardiovascular disease risk of underserved populations, demonstrating both the importance and difficulties of cross-sectoral partnerships and community engagement.
• Curran and colleagues’ article builds on the 2012 codification of hybrid effectiveness-implementation studies to conceptualize them as encompassing a broad range of designs beyond controlled trials, advising how study teams can integrate contextual knowledge and collaborative viewpoints to accordingly plan their hybrid effectiveness-implementation study.

For this Research Topic to truly serve as springboard for the next generation of hybrid studies, readers are encouraged to (i) carefully appraise the merits and limitations of the articles’ presented findings and suggested improvements and (ii) actively engage in forums of thought exchange to enhance hybrid effectiveness-implementation studies’ role in speeding the translation of research evidence into routine practice in health services at local, regional, national, and global levels.

